# The structure of the Ca^2+^-binding, glycosylated F-spondin domain of F-spondin - A C2-domain variant in an extracellular matrix protein

**DOI:** 10.1186/1472-6807-11-22

**Published:** 2011-05-10

**Authors:** Kemin Tan, Jack Lawler

**Affiliations:** 1 Midwest Center for Structural Genomics and Structural Biology Center, Biosciences Division, Argonne National Laboratory, Argonne, IL 60439, USA; 2 Division of Experimental Pathology, Department of Pathology, Beth Israel Deaconess Medical Center and Harvard Medical School, Boston, MA 02215, USA

## Abstract

**Background:**

F-spondin is a multi-domain extracellular matrix (ECM) protein and a contact-repellent molecule that directs axon outgrowth and cell migration during development. The reelin_N domain and the F-spondin domain (FS domain) comprise a proteolytic fragment that interacts with the cell membrane and guides the projection of commissural axons to floor plate. The FS domain is found in F-spondins, mindins, M-spondin and amphiF-spondin.

**Results:**

We present the crystal structure of human F-spondin FS domain at 1.95Å resolution. The structure reveals a Ca^2+^-binding C2 domain variant with an 8-stranded antiparallel β-sandwich fold. Though the primary sequences of the FS domains of F-spondin and mindin are less than 36% identical, their overall structures are very similar. The unique feature of F-spondin FS domain is the presence of three disulfide bonds associated with the N- and C-termini of the domain and a highly conserved N-linked glycosylation site. The integrin-binding motif found in mindin is not conserved in the F-spondin FS domain.

**Conclusion:**

The structure of the F-spondin FS domain completes the structural studies of the multiple-domain ECM molecule. The homology of its core structure to a common Ca^2+^- and lipid-binding C2 domain suggests that the F-spondin FS domain may be responsible for part of the membrane targeting of F-spondin in its regulation of axon development. The structural properties of the FS domain revealed in this study pave the way for further exploration into the functions of F-spondin.

## Background

**Figure 1:**
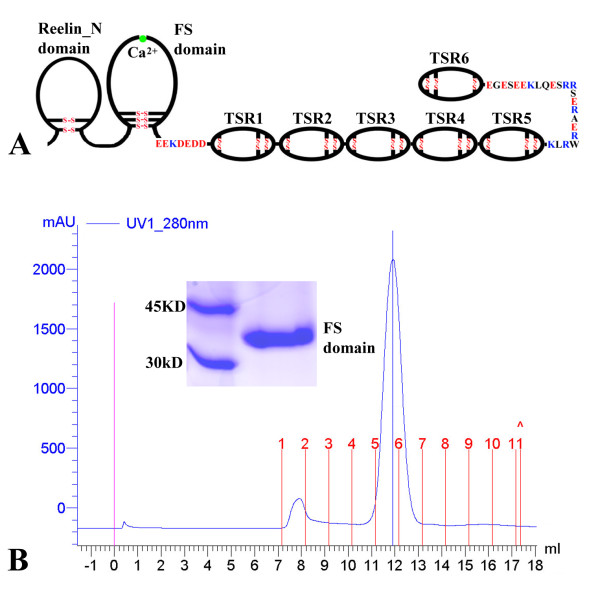
**The multiple domain structure of F-spondin and FS domain expression**. (A) Schematic diagram of the domain structure of F-spondin showing the disulfide bond patterns of each domain and highly charged linkers between FS domain and TSR1, and between TSR5 and TSR6. (B) Size-exclusion chromatography of the recombinant FS domain. The recombinant protein was first purified using Ni-affinity chromatography. In the following FPLC runs with a Superdex™ 75 column (GE Healthcare Life Sciences), six protein standards were used to calibrate the column (data not shown). In the run of FS domain, the molecular mass of the principal peak was calculated to be 22.7 kDa, which was smaller than the calculated molecular mass of 27.23 kDa that does not include vector-derived sequences and N-linked glycans. SDS PAGE of the FS domain revealed a single band with a molecular mass greater than 30 kDa (insert).

F-spondin was initially identified in the rat embryo floor plate[1], a ventralizing structure implicated in the control of neural cell patterning and axon growth in the developing vertebrate nervous system. The expression level of F-spondin is high in the floor plate at the time of axon extension[1]. Further studies have found that F-spondin plays an important role in the outgrowth of sensory[1], commissural[2] and hippocampal[3] neurons during development as well as in the migration of distinct somite domains to neural crest[4]. F-spondin is an extracellular matrix (ECM) protein with multiple domains, including an N-terminal domain (reelin_N domain)[5], an F-spondin (FS) domain and six thrombospondin type 1 repeats (TSRs) (Figure 1A). F-spondin, which is secreted by cells within the floor plate, is proteolytically processed into fragments that differentially bind to the floor plate cells or the basement membrane[6]. The portion that binds to the floor plate cells acts as a short-range repellent of commissural axons and prevents their migration into the floor plate. Other portions of F-spondin that includes the FS domain accumulate at the basement membrane and support growth cone migration[6]. The proteolysis of F-spondin and the coordinated interaction of the different fragments with the membrane of floor plate and the basement membrane provide a combinatorial guidance signal for commissural axons that cross the midline[6]. F-spondin is also highly up-regulated in injured peripheral nerves and it promotes outgrowth of sensory neurons[2]. An antibody against the FS domain blocks neurite outgrowth, indicating the FS domain plays an active role in axon regeneration[2]. This observation is largely in agreement with the fact that an F-spondin fragment that lacks the TSRs is sufficient to promote neuronal differentiation[7].

The expression of F-spondin indicates that it also functions outside the nervous system. F-spondin is expressed in various organs including the ovary, lung, kidney, small intestine, prostate[8] and testis[9]. Human and bovine F-spondins, initially called vascular smooth muscle cell (SMC) growth-promoting factor (VSGP), were first cloned from ovarian cDNA libraries, and bovine F-spondin was purified from adult bovine ovarian follicular fluid[8]. F-spondin may be a major factor for SMC proliferation in the ovary. *In vitro *experiments suggested that F-spondin may also contribute to the inhibition of angiogenesis via the functional blockade of endothelial integrin αvβ3, which plays important roles in angiogenesis[10]. It has been recently shown that F-spondin is a promoter for cementoblastic differentiation[11]. The molecule is up-regulated in osteoarthritis, promoting prostaglandin production, collagen degradation and proteoglycan synthesis reduction, probably via the activation of latent TGF-β[12].

The first identification of ligands for F-spondin came from the studies of the pathogenesis of Alzheimer's disease[13,14]. Amyloid precursor protein (APP) is a type I membrane protein. Its extracellular domain is initially cleaved by α- or β-secretases and subsequently by γ-secretase to secrete small peptides including Aβ40 and Aβ42, which are the primary components of the amyloid-β fibrils found in Alzheimer's disease. F-spondin interacts with the extracellular domain of APP, blocks APP cleavage by β-secretases, and eventually inhibits the production of the Aβ peptides by γ-secretase[13,14]. A working model suggested that F-spondin forms a heterotrimer assembly on the membrane with APP and apolipoprotein E receptor 2 (apoEr2) in which the reelin_N domain and the FS domain interact with APP and TSRs interact with apoEr2[14,15].

Recently, the structure of mindin FS domain has been reported[16]. Mindin is a member of the Mindin/F-spondin family[3,17] and an integrin-binding and pattern-recognition molecule for microbial pathgens[16,18,19]. In this paper, we present the crystal structure of human F-spondin FS domain. With the structures of the N-terminal (reelin_N) domain[5,20] and TSRs[21] of F-spondin characterized, the structure of FS-domain is the last to be determined and studied.

## Results and Discussion

### Overview of FS domain structure

**Figure 2:**
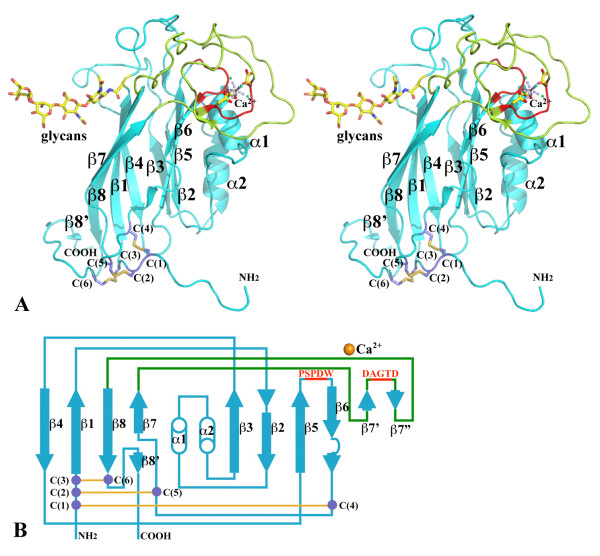
**The structure of the FS domain**. (A) A stereo view of ribbon drawings of the FS domain. The two highly conserved sequences [17], PSPDW (mostly on the β5-β6 loop) and DAGTD (on the unusually long β7-β8 loop) are colored in red. Ca^2+^-binding residues on the top of the FS domain, the three disulfide bonds at the bottom of the domain and glycans are drawn in stick format. (B) The topology scheme of the FS domain. Figure 2, 3 and 5 were prepared using the program Pymol[37].

**Figure 3:**
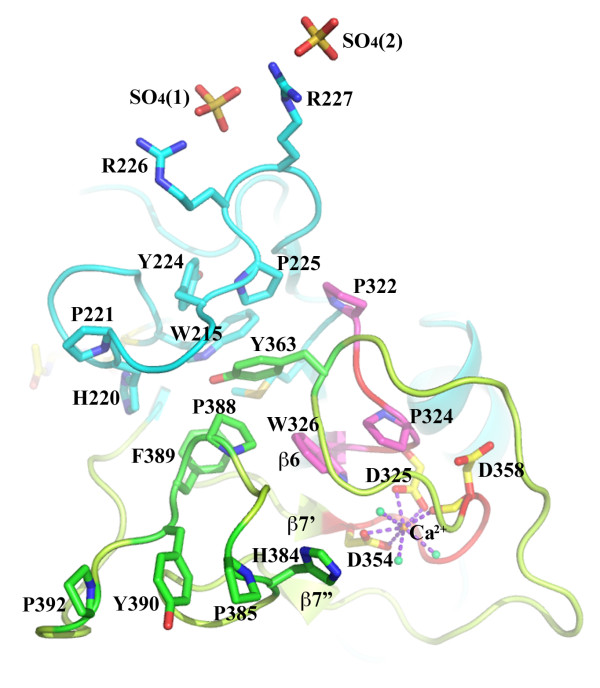
**The top of the FS domain**. Besides Ca^2+^-binding site, two of three SO_4_^2- ^groups (from crystallization buffer) identified in the structure are associated with R226 and R227 on one side of the top of the FS domain. The top of the FS domain is also rich in aromatic residues and prolines. The β7-β8 loop covers most the of FS domain on one side. When it turns at Y363, the sidechain of Y363 dips into a pocket containing aromatic residues and prolines, including P388 and F389. After the β7" short strand, four rings from H384, P385, Y390 and P392 are parallel to each other in a row. Additional aromatic residues from other loops are also found on the top of the domain, including W215, W231, P324, W326, P352, W353, Y363 and F383. All of these residues are either identical or highly conserved in the FS domains of other proteins (Figure 4). The role of these aromatic residues is unknown.

The core of the FS domain has an 8-stranded β-sandwich fold, with strands β4, β1, β8, and β7 forming one β-sheet and strands β3, β2, β5 and β6 forming the other sheet (Figure 2). The topology represents one of two types of C2 domain β-sandwich folds[22]. The first major variation of the FS domain, as compared to a common C2 domain, is its long linker (38 amino acids) between β2 and β3 strands, which actually forms two anti-parallel α-helices, packed against the β3/β2/β5/β6 sheet. The second variation is its long linker (51 amino acids) between the β7 and β8 strands. The linker first crosses over to the β3/β2/β5/β6 sheet and forms a short strand, β7' (W353D354) that is anti-parallel to the edge strand, β6 (Figure 2 and 3). It then runs above the β5-β6 loop and turns back at Y363. The linker forms a second short strand, β7" (R377-P378), by β7'. The addition of the two short strands is an expansion of the β3/β2/β5/β6 sheet to a 6-stranded sheet. Additionally, a part of the loop between β2 and α1 (G249-G250) runs in an anti-parallel fashion by the β7" strand, forming two mainchain-mainchain hydrogen bonds with the residue I376 right before β7". These two interactions (G249 N- I374 O and G250 O-I374 N) do not define another β strand.

**Figure 4:**
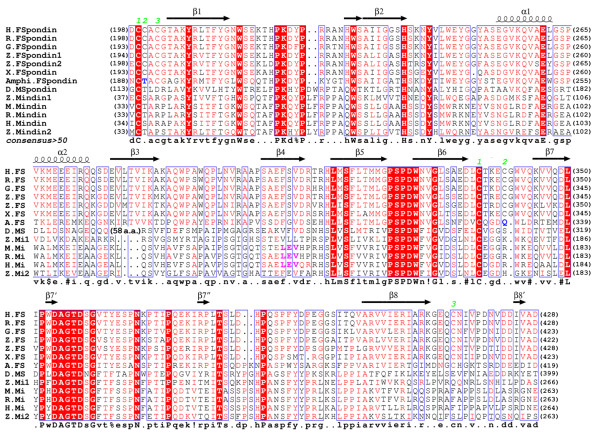
**Multiple sequence alignment of FS domains from proteins across different species**. Based on the FS domain structures of the two monomers, the secondary structures are indicated with arrows (β-strands) and coils (α-helices) above the appropriate sequences. Identical residues are highlighted in red. Disulfide bond forming cysteines are indicated with green numbers. Disulfide bond substitution, potential hydrogen bond forming residues (T190 and Q330) in amphiF-spondin are colored in blue. The integrin-binding LEV triplet found in human, mouse and rat mindins are highlighted in magenta. The sequences used in the alignment include human F-spondin (gi:110347423), rat F-spondin (gi:544353), chicken F-spondin (gi:45382337), *Xenopus *F-spondin (gi:544354), zebrafish F-spondin-1 (gi:18859413), zebrafish F-spondin-2 (gi:2529227), amphiF-spondin (gi:3319874), *Drosophila melanogaster *F-spondin (gi: 19922086), human mindin (gi: 52783469), mouse mindin (gi: 52783454, rat mindin (gi: 52783424) and zebrafish mindin-1 and -2 (gi: 82069286 and gi: 82069288). The alignment was generated with the program MultAlin[38] and ESPript[39]. In *Drosophila melanogaster *F-spondin (D. M-spondin), the large, likely unstructured, Ser, Gly, Thr and Ala-rich insertion (VQMQLQSQMQAGKSPSGGISSGTTSFNATAASTATPTGGSGGSGGSGGSGGTGTTTAE) between α3 and β2 is not shown in the alignment.

The residues contributing to the hydrophobic core of the FS domain are A205, Y207, L209 and F211 of the β1 strand, I234 of the β2 strand, F305 and V307 of the β4 strand, M314 and F316 of the β5 strand, as well as A401, V403 and I405 of the β8 strand (Figure 4). Based on the sequence conservation of these residues among FS domains of F-spondins, mindins, M-spondin as well as AmphiF-spondin, we predict that a C2 domain core structure should be conserved in these proteins.

### Disulfide bond pattern

The three consecutive cysteines (C199, C200 and C202) before the β1 strands form three disulfide bonds with C336 and C340 from the β6-β7 loop, and C415 after the β8 strand at the C-terminal region, respectively (Figure 2). None of these disulfide bonds is located within the core of the β-sandwich domain. They are all at the bottom of the FS domain and are associated with N- and C-termini, especially the N-terminus, where they seem to help define and stabilize the globular FS domain (Figure 2). The construct for protein expression begins from V191. In the electron density maps, D198 is the first N-terminal residue visible while the other seven residues together with 6 vector-derived residues were disordered in the structure. Interestingly, after the β8 strand, about 13 residues between C415 and D428 are clear in the electron density maps. Two of them, I425 and V426, form a short strand (β8') parallel to β8, providing structural stability to the C-terminal loop region after C415.

There is a noticeable difference in the number of disulfide bonds in the FS domains of F-spondin and mindin. Early understanding of the FS domain structure was based on two very conserved sequence repeats, fs1 and fs2, which were suggested to represent two individual domains[17]. However, an analysis based on the number of cysteine residues and their distribution actually suggested a disulfide bond pattern that was not in agreement with the early prediction. In the F-spondin FS domain, there are six cysteines, designated C(1) to C(6). C(1-3) are in the fs1 repeat, C(4-5) are in the fs2 repeat and C(6) is in a region after fs2. In mindin, only C(1) and C(4) exist, suggesting a possible disulfide bond C(1)-C(4) linking fs1 and fs2. In AmphiF-spondin, C(2) and C(5) are both missing and are replaced by a pair of hydrogen bond forming residues, T190 and Q330 (Figure 4). This implies the second disulfide bond C(2)-C(5) is also between fs1 and fs2 in other proteins. The remaining cysteine (C(3)) of fs1 likely forms the third disulfide bond with C(6) after fs2. These results are consistent with the conclusion that fs1 and fs2 are most likely within a single domain. The functionality and the evolution of the varied disulfide bond patterns are unknown. A phylogenetic analysis using the alignment in Figure 4 as an input suggests that FS domains of F-spondins and mindins have evolved separately from their common ancestor[23]. The result largely agrees with the observation that the numbers and the positions of the exons that cover FS domains in genomic DNAs are different between F-spondins and mindins.

### C-termini of FS domains

**Figure 5:**
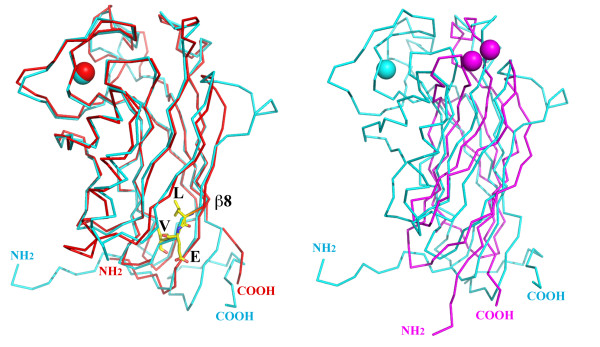
**The structural alignment of F-spondin with mindin and a typical C2 domain**. (A) An alignment of FS domains of F-spondin (in cyan) and mindin (in red). Ca atoms are shown as spheres. The integrin-binding LEV triplet of human mindin is drawn in stick format. Only the C-terminal β8 stand is labeled. (B) An alignment of the FS domain of F-spondin (in cyan) with the N-terminal Ca-dependent lipid-binding/C2 domain of cytosolic phospholipase A2 (in magenta, PDB code:1CJY)[27].

The C-terminal region of FS domains is one of the most diversified regions in the amino acid sequences (Figure 4). The location of the C-terminus of F-spondin or mindin is different according to the structures of their FS domains. In the construct of mindin[16], the C-terminus was truncated at R223, which is equivalent to E413 of F-spondin. In the structure reported here, E413 is before C415 and on the structured C-terminal loop between the β8 and the β8' strands. In mindin, there is no disulfide bond tethering its C-terminal region to its N-terminus. The end of the β8 strand is L218 and the following residue R219 starts to peel off from the mindin FS domain (Figure 5A), resulting in disordering of most of the other C-terminal residues of the construct[16]. In F-spondin, residue A409, the equivalent of R219 in mindin, is the third residue from the end of the β8 strand (Figure 4). The C-terminal loop region observed in F-spondin seemingly extends the C-termini of the FS domain to at least V426 (Figure 2). We note that exon 10 of human F-spondin genomic DNA encodes the C-terminal region of the FS domain, from G412 to E436. It is possible that the C-terminus of F-spondin FS domain extends much beyond the disulfide bond formed by C415. However, we can't exclude the possibility that the C-terminal structured region (from N416 to V426) results from molecular packing in the crystal.

### N-linked glycosylation, Ca^2+^- and SO_4_^2- ^-binding sites

There is one N-linked glycosylation site (N214) at the end of the β1 strand in the FS domain (Figure 2A). The common pentasaccharide core of the linked glycans is visible in the electron densities. Two branched mannose residues were not built in the final model due to their weak densities. Like other proteins that we have expressed in *Drosophila *S2, the N-linked glycans are not fully processed[5,24]. The post-translational modification in the FS domain is conserved only in F-spondins while in mindins only zebrafish mindin-1 and -2 FS domains are glycosylated, at different sites (Figure 4).

Associated with the short strands at the top of the FS domain, there is a solvent-accessible Ca^2+^-binding site, formed from D325 (from β5-β6 loop), D354 (from β7' short strand) and D358 (right after β7' short strand). The site also contains three coordinating water molecules in an approximately octahedral coordination geometry (Figure 2 and 3). All three aspartic acids are from the two highly conserved and characteristic FS domain "repeats", P322SPDW and D354AGTD (in F-spondin) [17]. Both D325 and D354 interact with the Ca^2+ ^ion as bidentate ligands involving both carboxyl groups with bond distances of 2.49 Å/2.53 Å and 2.38 Å/2.46 Å, respectively. Interestingly, D358 interacts with the Ca^2+ ^ion with only its mainchain carbonyl group (2.33 Å) though the residue is part of a highly conserved motif. All three aspartic acids and three coordinating waters (2.47 Å for all) are part of an extensive hydrogen bond network. The geometry of the metal-binding site, including bond distances, coordination[25] as well as the similar B-factors of the Ca^2+ ^ion (16.2 Å^2^) and its surround protein atoms (~15.5 Å^2^), support the metal ion assignment.

Three sulfate groups from the crystallization buffer that included 0.2M lithium sulfate monohydrate have also been identified in the structure (Figure 3). One of them is inside a pocket near the bottom of the domain (not shown in the figure). The other two sulfate groups, labeled as SO_4_(1) and SO_4_(2) in Figure 3, are associated with two consecutive arginines, R226 and R227, on one side of the top of the FS domain. The SO_4_(1) occupies a special position with 2-fold crystallographic symmetry, and binds the residues R226 and R227, and the same residues from an adjacent, symmetry-related FS molecule.

### Structural homologues

The primary sequence identity of the FS domains between F-spondin (human, a.a.198-428) and mindin (human, a.a.34-264) is 35.9%. A secondary-structure matching (SSM) superposition[26] of two molecules yields a core root mean square deviation (rmsd) of 1.14 Å with 200 residues from each aligned (Figure 5A). In the alignment, there are eight gaps, all in loop regions with the major difference (an insertion of six residues in F-spondin) occurring before the β4-strand and creating a protrusion from the edge of the β4/β1/β8/β7 sheet. The protrusion is accompanied by the N-linked glycans extended from the end of the neighboring β1-strand as described earlier.

A Dali structural homology search (http://www2.ebi.ac.uk/dali/) using the F-spondin FS domain found a large number of fits (Z ≤ 5.8 and rmsd ≥ 3.3 Å), including the N-terminal Ca^2+^-dependent lipid-binding/C2 domain of cytosolic phospholipase A2 (PDB code:1CJY) [27]. In the structural alignments of the FS domain with these C2 domains, only the core of the FS domain (commonly about 110-120 residues) can be superimposed, indicating that the FS domain represents a derivative of a common C2 domain (Figure 5B). Though helices are commonly found within the linker between two strands in C2 domain structures, the two nearly anti-parallel helices within the loop between the β2 and β3 strands and the unusual long loop between the β7 and β8 strands found in the FS domain are unique to FS domains only.

### Implication of Potential Interactions

Membrane targeting is an important part of axon guidance of the proteolytic fragment of F-spondin that includes the FS domain. The unexpected identification of a C2 domain homology with the FS domain and the presence of Ca^2+^- and SO_4_^2-^-binding sites implies potential roles of this domain in the interaction of F-spondin with membrane and/or membrane associated molecules.

The C2 domain is the second most abundant lipid binding domain behind the pleckstrin homology (PH) domain[28,29]. Though the C2 domain can fold individually with a highly conserved common β-sandwich core, it seems to be a modular domain that is found in a large number of multiple domain proteins involved in signal transduction or membrane trafficking. Most of the C2 domain-containing molecules that have been studied to date are either soluble proteins or membrane proteins. The FS domain structures of mindin and F-spondin represent the first examples of structures from ECM proteins that contain a C2-domain or rather a C2-domain derivative. The individual C2 domains commonly participate in Ca^2+^-dependent membrane binding in Ca^2+^-mediated cell processes.

In most cases, the membrane-binding and/or lipid binding of C2 domains is dependent on the Ca^2+^-binding site(s), though the number of Ca^2+ ^binding-sites and their positions at the top of the domains are variable[28]. Ca^2+ ^ions can (1) function as an electrostatic switch for favorable ionic interaction with the anionic membrane, (2) form a bridge between the C2 domain and anionic phospholipids, or (3) induce conformational changes that lead to protein-membrane interactions[28]. The exact methods for the C2 domain interaction with membranes, including lipid selectivity, may be dependent on the number of Ca^2+^-binding sites and the conformation of each Ca^2+^-binding site. Additionally, protein-membrane associations mediated by divalent metals, especially Ca^2+ ^ions, have been reported in many other proteins, including vitamin-K-dependent proteins, annexins and pentraxins[30].

The presence of a sulfate group from crystallization buffer in a crystal structure could be an indicator of a binding site of an anionic group of a potential ligand, such as heparin (with sulfate group) and lipid (with phosphate head group). The arginines, R226 and R227, on one side of the top of the FS domain could contribute to a binding site for the phosphate head group of a lipid. It is interesting that R226 is conserved between F-spondins and mindins while R227 in mindins is replaced by a proline (Figure 4). Heparin-binding has been demonstrated for F-spondin's N-terminal reelin_N domain[5], which is adjacent to the FS domain (Figure 1A). The potential for the two consecutive arginines R226 and R227 of the FS domain to bind heparin in synergy with the reelin_N domain remains to be explored. The presence of two sulfate groups, especially SO_4_(1) at a crystallographic special position, could be an artifact of molecular packing. The heparin-binding, or more generally glycosaminoglycan (GAG)-binding, implies another potential pathway for targeting the membrane through the interaction with membrane-associated GAGs such as those from transmembrane proteoglycans.

## Conclusions

The structure of F-spondin FS domain, together with mindin FS domain, represents the first examples of C2 domain-like structures in ECM proteins. Although the two FS domains have similar overall structures, they have evolved separately from their common ancestor. The integrin-binding motif found in mindin is not conserved in the F-spondin FS domain. The F-spondin FS domain is unique with a conserved N-glycosylation site and three disulfide bonds, which may play critical roles in protein folding and stability as well as defining domain boundaries. The homology of its core structure to a common Ca^2+^- and lipid-binding C2 domain suggests that the F-spondin FS domain may be responsible for part of the membrane targeting of F-spondin in its regulation of axon development. The structure of the F-spondin FS domain completes the structural studies of the multiple domain ECM molecule and its properties revealed in this study pave the way for further exploration into the functions of F-spondin.

## Methods

### Preparation of the Recombinant FS domain

A cDNA pool was prepared from a mixture of human spleen, placenta and liver poly(A)+ RNA (Stratagene, La Jolla, CA) using a reverse transcription kit (Gibco-BRL, Gaithersburg, MD). A recombinant version of the human F-spondin FS domain (amino acids V191-K434) was prepared by PCR using the cDNA pool as the template. The FS domain was prepared using the forward primer fs102f (5'-GAT GAT CCC GGG GTG ACT GAC AAA CCC ATC-3') and the reverse primer fs104r (5'-ATG ATG ACC GGT TTT CTC TTC TGG AGC CAG-3'). The PCR products were sequenced and cloned between the *Sma*I and the *Age*I sites of the vector pMT/BiP V5-HisA (Invitrogen, Carlsbad, CA) for expression in *Drosophila *Schneider 2 (S2) cell. The recombinant proteins include the vector-derived sequence RSPWPG at the N-terminal and the sequence TGHHHHHH at the C-terminal.

The vector transfection and cells selection were performed following the protocol from Invitrogen, catalog no. R690-07. The methods for FS domain expression and purification were described previously[31]. The protein was further purified by FPLC in buffer containing 200 mM NaCl and 20 mM HEPES at pH 7.8 with sodium as the counter ion. The apparent molecular weight of the eluted FS domain was estimated to be 22.7 kDa based on the calibration of the column with known proteins, indicating the domain exists as a monomer in solution (Figure 1B).

### Crystallization and X-ray Diffraction Data Collection

The purified protein was concentrated to about 30 mg/ml. The crystallization was carried out with sitting drop vapor diffusion technique. Protein crystals grew from the buffer containing 0.2 M lithium sulfate monohydrate, 25% (w/v) PEG 3350 and 0.1 M HEPES at pH 7.5. The crystals had a hexagonal form with a dimension of about 60 μm on edge. The crystals were then treated with a cryoprotectant (10% glycerol in crystallization buffer) and flash frozen in liquid nitrogen.

One X-ray diffraction data set was collected from a frozen crystal at 100K at the 19ID beamline of the Structure Biology Center at the Advanced Photon Source, Argonne National Laboratory. The data set was processed using the program suite HKL3000[32] (Table 1). An X-ray fluorescence spectrum of the crystal was measured with the primary beam energy of 12.659 keV. Only two peaks, from the primary beam and Compton scattering, respectively, were recorded (data not shown).

**Table 1:** Crystallographic Statistics

Data Collection	*Native*
Space group	*P*622

Unit Cell (Å)	*a *= *b *= 120.24, *c *= 86.36

MW Da (residue)	27230(244)^1^

Mol (AU)	1

Wavelength (Å)	0.97940

Resolution (Å)	1.95

Number of unique reflections	27486

Redundancy	10.1 (6.4)^2^

Completeness (%)	99.9(100.0)^2^

R_merge _(%)	12.6 (64.6)^2^

I/σ(I)	23.4 (3.4)^2^

**Structural Refinement**	

Resolution (Å)	1.95-33.5

Reflections (work/test)	25965/1374

R_crystal_/R_free _(%)	18.0/21.4

Rms deviation from ideal geometry Bond length (Å)/angle (°)	0.009/1.135

No. of atoms (Protein/HETATM)	1911/402

Mean B-value (Å^2^) (mainchain/sidechain)	19.90/20.70

Ramachandran analysis (%)^3 ^Residues in favored regions/in allowed regions.	97.10/ 100

Protein Geometry MolProbity score^3^	0.79

^1 ^Not including cloning artifact and His tag.

^2 ^(Last resolution bin, 1.95-2.00 Å).

^3 ^From MolProbity[36].

The F-spondin FS domain structure was determined with molecular replacement method using the program MolRep[33]. One monomer from the mindin FS domain structure (PDB code:3D34) was used as a search template. After sequence substitution and manual model building using the program Coot[34], structural refinement was performed using the program Refmac[35] (Table 1). The final model includes residues from T192 to D428, N-linked glycans (up to the first mannose moiety) attached to the residue N214, one Ca^2+ ^ion, three sulfate groups from crystallization buffer and 344 water molecules. The quality of the structure was validated with MolProbity[36] (Table 1).

The coordinates and structure factors of the human F-spondin FS domain have been deposited in the Protein Data Bank with accession code 3Q13.

## Authors' contributions

KT conceived of the study and carried out cloning, cell culture, protein purification and crystallization, X-ray diffraction data collection and structural determination, structural analysis and manuscript preparation. JL participated in the study design and coordination, sequence analysis and construct design, structural interpretation and manuscript preparation. All authors read and approved the final manuscript.
